# Supplementary material for: Microstructure and Oxygen Evolution Property of Prussian Blue Analogs Prepared by Mechanical Grinding
Source: Nanomaterials (Basel). 2023 Aug 30;13(17):2459. doi: 10.3390/nano13172459 (PMC10489616; doi:10.3390/nano13172459)
Supplement: Supplementary file 1 [file nanomaterials-13-02459-s001.zip › nanomaterials-2563224-supplementary.pdf]

nanomaterials

Supporting Information

Microstructure and Oxygen Evolution Property of Prussian Blue Analogs Prepared by Mechanical Grinding

Abhishek Meena [1], Chinna Bathula [2], Mohammad Rafe Hatshan [3], Rama Subbareddy Palem[4] and Atanu Jana [1,*]

[1] Division of Physics and Semiconductor Science, Dongguk University-Seoul, Seoul 04620, Republic of Korea; pakar.abhishek@gmail.com
[2] Division of Electronics and Electrical Engineering, Dongguk University-Seoul, Seoul 04620, Republic of Korea; cdbathula@dongguk.edu
[3] Department of Chemistry, College of Science, King Saud University, P.O. Box 2455, Riyadh 11451, Saudi Arabia; mhatshan@ksu.edu.sa
[4] Department of Medical Biotechnology, Dongguk University, 32 Dongguk-ro, Ilsandong-gu, Goyang, Gyeonggi 10326, Republic of Korea; palemsubbareddy@gmail.com
* Correspondence: atanujanaic@gmail.com; Tel.: +82-1068502422

Index

SI. No.	Content	Page No.
1	Characterization	**2**
**Table S1.**	Sample name and its precursors	**2**
**Figure S1.**	Mechanochemical synthesis of Prussian blue (PB) and Prussian blue analogues (PBAs) compounds.	**3**
**Figure S2.**	High-resolution XPS of **S1**. (a) Overall, XPS survey spectra. (b) Fe 2p. (c) N 1s. (d) C1s. (e) N (e) O1s.	**4**
**Figure S3.**	High-resolution XPS of **S2**. (b) Fe 2p. (c) Co 2p. (d) O1s. (e) N 1s(e) C1s	**4**
**Figure S4.**	OER polarized curves for **S1**, **and S3** in 1 M KOH.	**5**
**Figure S5.**	Gram-scale synthesis of electrocatalyst **S3.**	**6**
**Figure S6.**	SEM of electrocatalyst **S3** after OER**.**	**6**
**Figure S7.**	(a-f) The capacitive currents at the potential window as a function of different scan rates (20, 40,60,80 and 100) and the linear slope corresponds to the double-layer capacitance (C_{dl}).	**7**
**Figure S8.**	(a) ECSA normalized the current density of the S1, S2, S3 catalysts. (b) S3 catalyst long-term stability test analysis at a constant density of 100 mA cm $^{-2}$.	**7**

Characterization Methods

Scanning electron microscopy (SEM) was carried out with a field emission SEM (FE-SEM, Nova 230, FEI) with an accelerating voltage of 10kV. The High-power X-ray Diffraction patterns were obtained with D/MAX2500V/PC diffractometer, Rigaku using Cu-rotating anode X-ray. PXRD was collected using the K-alpha model, ThermoFisher. During the measurement, the Bragg's diffraction angle (2θ) was set to 10-80°, and the scan rate was 2°/minute. Fourier Transformed Infrared spectra were collected in FTIR (670-IR, Varian) with an attenuated total reflection detector. The optical diffuse reflectance spectra of all samples were recorded using a Cary 5000 UV-Vis-NIR Spectrophotometer (Agilent) with an integrated sphere in diffuse-reflectance mode and then converted to absorbance. Thermal gravimetric analysis was obtained with SDT Q600. During the measurement, the temperature was set to 50-890°, and the heating rate was 10°/minute heat under 100ml/minute nitrogen flow.

The electrochemical measurements were done on the VSP instrument (Bio Logic Science Instruments, Inc.) at room temperature in 1 M KOH. Ni foam was cleaned by ultrasonication for 5 min in HCl solution to remove the surface impurities, followed by washing with absolute ethanol and deionized water for 10 min. A three-electrode system was used for electrochemical measurements: reference electrode: Hg/HgO electrode, counter electrode: Pt wire, and working electrode: an NF substrate electrode). All potentials were converted to the potential of a reversible hydrogen electrode (RHE). The catalyst ink for the working electrode (NF) was made using the following method: 5 mg of catalyst was added to a mixture of 240 μl of ethanol and 440 μL of deionized water with 20 μL of Nafion (5 wt%), and the solution was sonicated for 30 min. Subsequently, 140 μL of catalyst ink was dropped onto the surface of the NF electrode, followed by drying at room temperature to reach a catalyst loading of 1 mg cm^{-2}. The iR correction was applied to all the electrochemical data reported. LSV data were obtained at a scan rate of 2 mV s^{-1}. Electrochemical impedance spectroscopy measurements (EIS) of the samples are performed in the frequency range of 10 kHz–10 mHz with an AC voltage of 10 mV in 1 M KOH solution.

Table S1. Sample name and its precursors.

Sample name	Precursor 1	Molar ratio	Precursor 2	Formula
**S1**	$FeCl_3$	4 : 3	$K_4[Fe(CN)_6]$	$Fe_4[Fe(CN)_6]_3$
**S2**	$CoCl_2$	3 : 2	$K_3[Fe(CN)_6]$	$Co_3[Fe(CN)_6]_2$
**S3**	$NiCl_2$	2 : 1	$K_4[Fe(CN)_6]$	$Ni_2[Fe(CN)_6]$

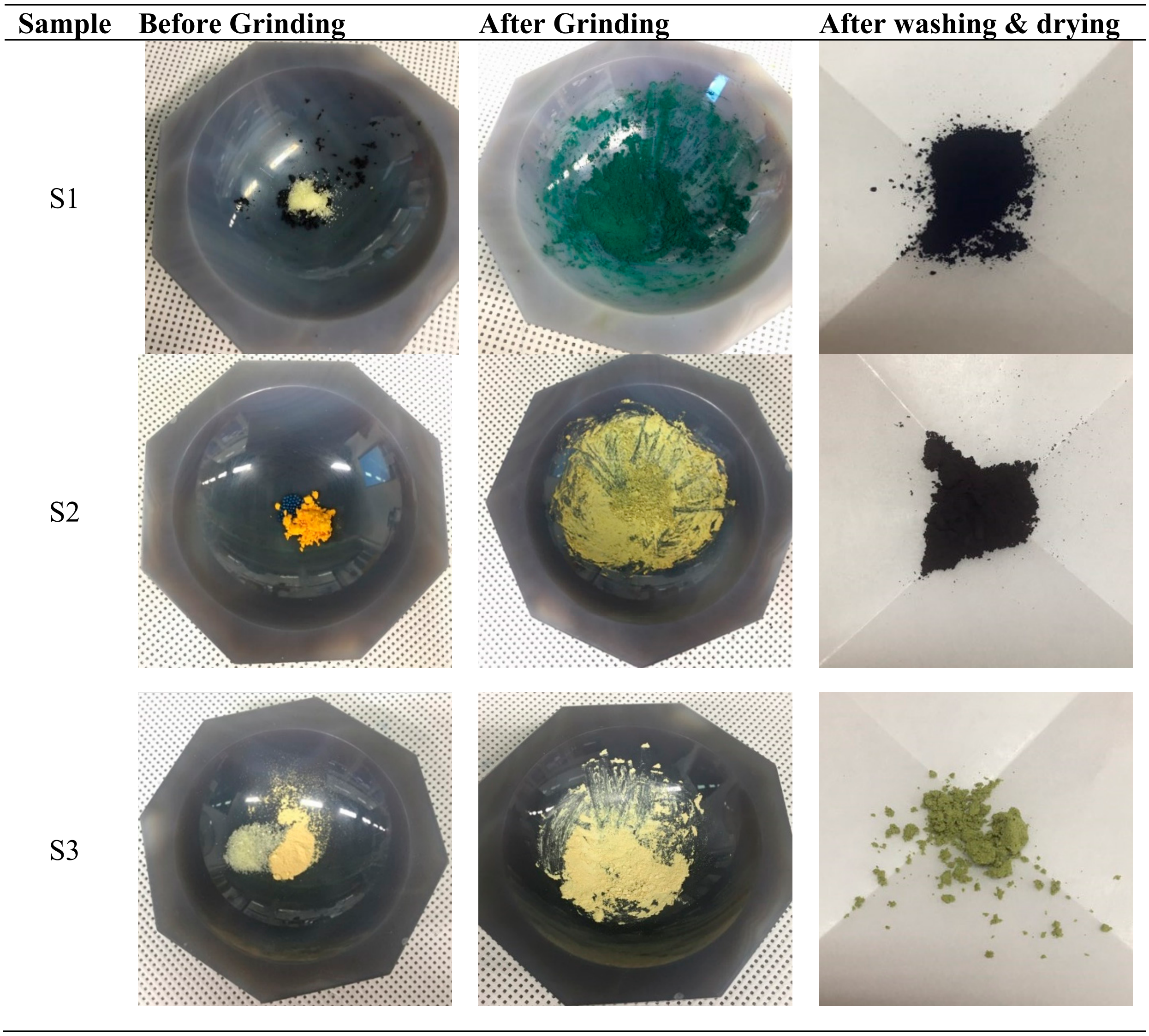

Figure S1. Mechanochemical synthesis of Prussian blue (PB) and Prussian blue analogues (PBAs) compounds.

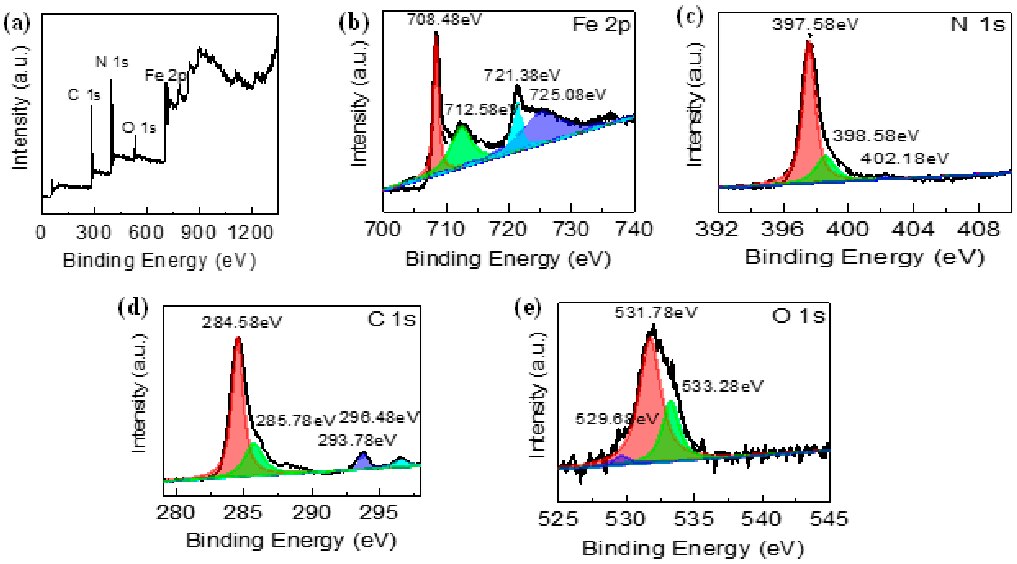

Figure S2. High-resolution XPS of **S1**. (a) Overall, XPS survey spectra. (b) Fe 2p. (c) N 1s. (d) C1s. (e) O1s.

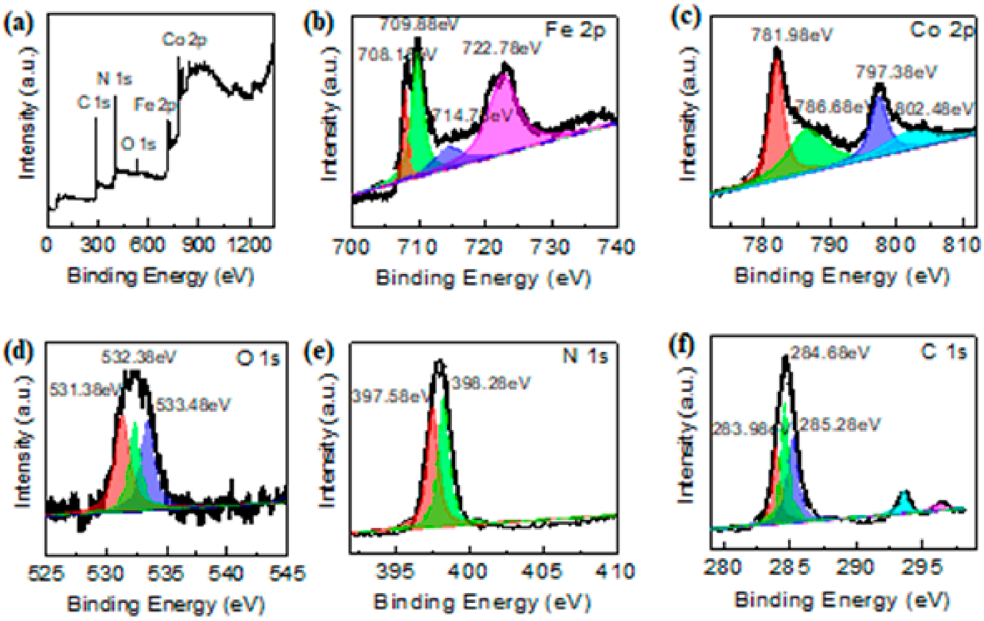

Figure S3. High-resolution XPS of **S2**. (a) Overall, XPS survey spectra. (b) Fe 2p. (c) Co 2p. (d) O1s. (e) N 1s(f) C1s.

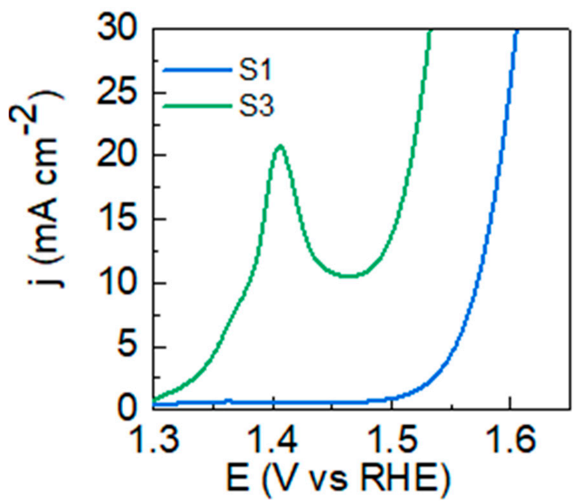

Figure S4. OER polarized curves for **S1, and S3** in 1 M KOH.

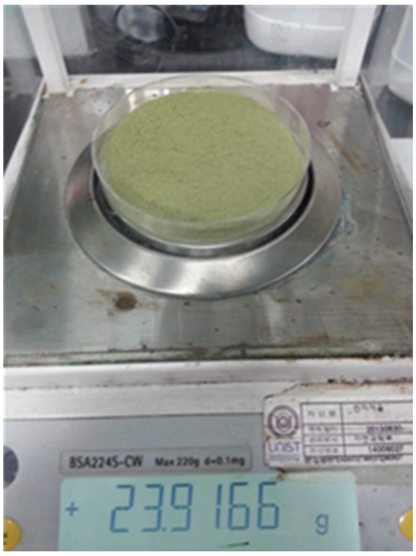

Figure S5. Gram-scale synthesis of electrocatalyst **S3.**

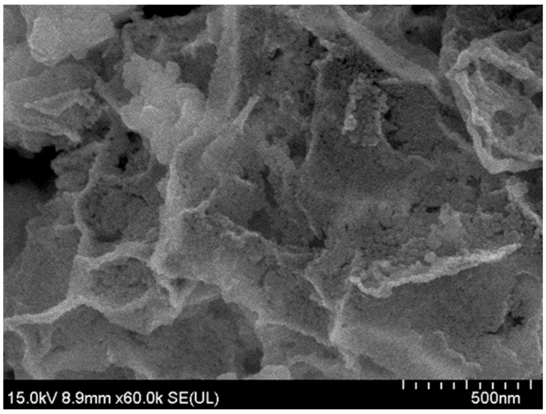

Figure S6. SEM of electrocatalyst **S3** after OER**.**

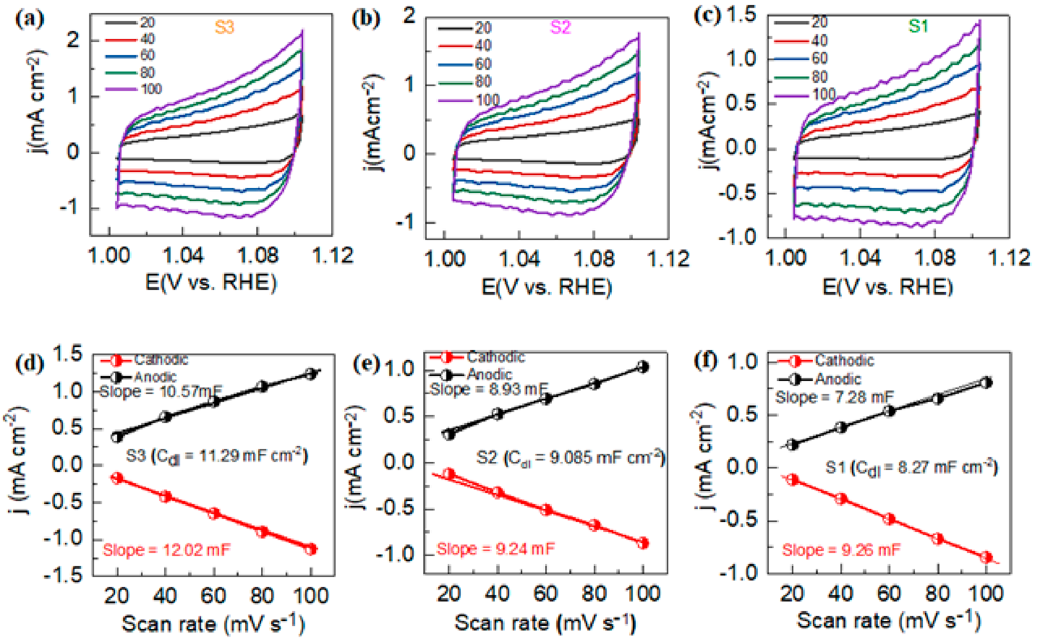

Figure S7. (a-f) The capacitive currents at the potential window as a function of different scan rates (20, 40,60,80 and 100) and the linear slope corresponds to the double-layer capacitance (C_{dl}).

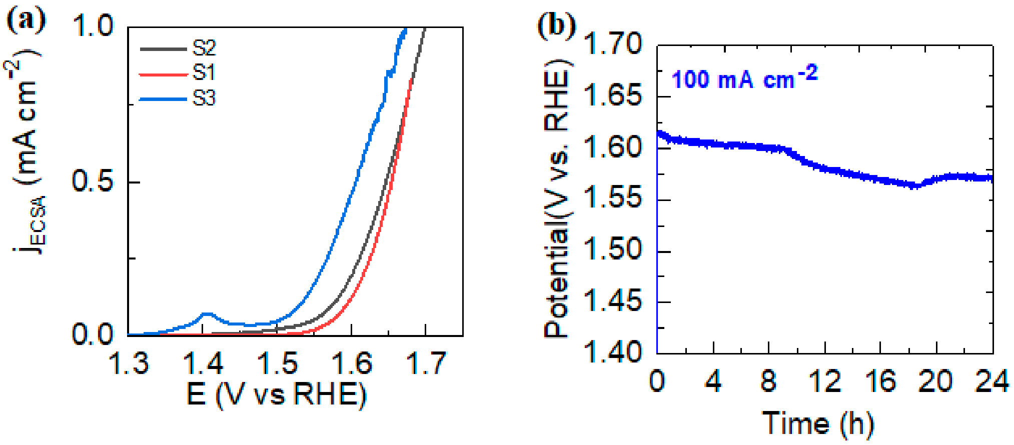

Figure S8. (a) ECSA normalized the current density of the S1, S2, S3 catalysts. (b) S3 catalyst long-term stability test analysis at a constant density of 100 mA cm $^{-2}$.
